# ‘PICO-D Management’; a decision-aid for evidence-based chiropractic education and clinical practice

**DOI:** 10.1186/s12998-016-0130-y

**Published:** 2016-12-12

**Authors:** Lyndon G. Amorin-Woods, Barrett E. Losco

**Affiliations:** School of Health Professions, Discipline of Chiropractic 90 South St Murdoch, Perth, 6150 Western Australia

**Keywords:** Algorithms, Chiropractic, Evidence-based practice, Practice guidelines

## Abstract

**Background:**

Various models and decision-making aids exist for chiropractic clinical practice.

**Results:**

“PICO-D Man” (Patient-Intervention-Comparator-Outcome-Duration Management) is a decision-aid developed in an educational setting which field practitioners may also find useful for applying defensible evidence-based practice. Clinical decision-making involves understanding and evaluating both the proposed clinicalintervention(s) and the relevant and available management options with respect to describing the patient and their problem, clinical and cost effectiveness, safety, feasibility and time-frame.

**Conclusions:**

For people consulting chiropractors this decision-aid usually requires the practitioner to consider a comparison of usual chiropractic care, (clinical management including a combination of active care and passive manual interventions), to usual medical care usually including medications, or other allied healthmanagement options while being mindful of the natural history of the persons’ condition.

## Background

Most existing models of clinical decision-making involve making a diagnosis and satisfying oneself that the patient is in the *‘right place at the right time’* [1]. Patient management in the contemporary health care environment is expected to be ‘evidence-based’ and suggests that patient outcomes are enhanced when their management is guided by the best available evidence [2, 3]. Furthermore it appears that when patient care is evidence based there is the potential for cost savings [4–6].

Undergraduate students are being trained in EBP [7], however it has been documented that it can be difficult to establish EBP amongst various professions, including chiropractic [8]. A number of potential barriers to the adoption of EBP in clinical practice have been identified and include; time restrictions, limited access to research studies, poor confidence in skills to identify and critically appraise research, and inadequate support [9–12]. A recent scoping review of chiropractors noted EBP gaps in the areas of assessment of activity limitation, determination of psychosocial factors influencing pain, general health indicators, establishing a prognosis, and exercise prescription. Chiropractors generally believe EBP and research to be important however use of EBP and guideline adherence varies widely [13]. There remain significant paradigmatic and cultural barriers in chiropractic along with other CAM professions to EBP; obstacles beyond merely the practical or knowledge deficiencies [14].

This paper seeks to simplify the clinical application of EBP by providing a clinical decision-aid. We use the example of acute or subacute low back pain presentation, since by far, the majority of patients that present to any chiropractic practice do so with spinal pain; be it labelled acute, subacute, chronic, non-specific, biomechanical or non-malignant [15]. It is important chiropractors realise the importance of adherence to clinical practice guidelines since spinal disorders are consistently within the top ten of the most expensive health care presentations [16, 17], thus some health system administrators are beginning to actually *require* practitioners to practice within clinical frameworks regardless of their profession particularly when third party payers such as insurers are involved [18].

Here we set out to expand on previously published models for clinical decision-making [19–21]. We feel since the one we present has been useful in an educational context it may assist field practitioners as well.

Models using diagnosis based decision-making must be tempered by the recognition that even experienced clinicians may be unaware of the correctness of their diagnoses at the time they make them [22–24]. Hoffman et al [21] presented a framework involving an active discourse between practitioner and patient which follows; *“What will happen if we wait and watch? What are the test or treatment options? What are the benefits and harms of these options? How do the benefits and harms weigh up for this patient? Do both patient and practitioner have enough information to make a choice”* [21]? Clinical decision support aids including the one we present assume and ensure use of best available evidence and patient-specific information to enhance patient care. They may encompass computerised alerts and reminders; clinical guidelines; condition-specific order sets; focused patient data reports and summaries; documentation templates; diagnostic support, and contextually relevant reference information [25].

Evidence based practice (EBP) aims to facilitate the practitioner’s clinical decision making process [26] and is based upon the premise that patient management should be guided by methodologically robust research findings [27–30]. The ‘three pillars’ or ‘three legged stool’ [28, 31] of evidence-based practice constitute the philosophical foundation of our model; effectively a ‘social constructivist’ or ‘participatory’ paradigm where clinical reality is ‘constructed’ by the participants; clinician and patient engaging throughout the course of clinical encounters and the care journey [32]. Clinicians should actually incorporate knowledge from 5 distinct areas into each management decision: empirical evidence, experiential evidence, physiologic principles, patient and professional values, and system features. The relative weight given to each of these areas is not predetermined, but varies from case to case [33]. It is important to remember everything ‘starts and ends’ with the patient. In our view this is a pragmatic, defendable stance reflective of sensible clinical practice recognising that the very application of EBP itself requires clinician expertise.

All clinicians must be mindful to practice ethically and competently within their own legally allowed *scope* of practice. For chiropractors this role is best described as primary *contact* rather than primary *care* [34, 35]. The clinicians’ own education, experience and specific expertise, including training at undergraduate and postgraduate level must underpin all clinical decision-making.

We suggest clinicians firstly to refer to evidence-based guidelines (EBGs) or consensus clinical practice guidelines (CPGs) relating as closely as possible to the problem of concern in their individual patient. ‘Best evidence’ is contained in a clinical guidelines, thus the clinician may be reasonably confident a robust process has been followed in assessing available evidence to achieve consensus by expert panels [36]. Clinical practice guidelines are a useful resource for clinicians as they preclude the clinician having to access all the literature, while protecting clinicians from ‘selective citation’. However, *clinical context*; does the evidence help one care for *this* patient, is then always a ‘value call’ for the clinician [37, 38].

The strength of a recommendation in a guideline reflects the extent of confidence that desirable effects of an intervention outweigh undesirable effects [39, 40], the strength of recommendations are determined by the balance between desirable and undesirable consequences of alternative management strategies, quality of evidence, variability in values and preferences, and cost [30, 39, 41].

Modifying this guideline ranking format to be relevant to patient choices could also be useful when providing a patient with their treatment/management options; *“Will it help?”* 1) *Probably*: thus most people in the same situation would choose the recommended course of action and only a small proportion would not, 2) *Possibly*: most people in the same situation would want the recommended course of action, but many would not, 3) *Maybe*: some people would choose the option but many would not, 4) *Unlikely*: some people may choose the option but most would not. Thus patient consent can be obtained in the context of probability, predictability and reliability of an outcome.

When there is inconclusive non-favourable evidence patients should be advised that this treatment is likely *not* to be effective and more effective treatments should be recommended where available. Where findings are reported as high and moderate quality negative evidence, patients should be actively advised *against* the use of this treatment and a more effective alternative should be recommended where available.

Patients’ expectations, goals, values and choices as components of EBP are important drivers of health care systems and technology developments [42, 43]. It remains critical that the priority of the patient’s right and ability to choose health care that suits their world view and personal preference is not compromised, so long as these choices are informed and reasonable and are not made as a result of coercion, deception or indefensible claims [44, 45]. Patients have questions; *“What is wrong with me?” “Can you help?” “Is what you do safe?” “What are my options?” “What will happen if I do nothing?” “How much will it cost?” “How long will it take?”* [46]. There are several additional questions the clinician should also ask themselves on behalf of the patient; *“What else could it be?” “Is there anything that doesn’t fit?”* and *“Is it possible there is more than one problem?”* The patient may well in the chiropractors’ opinion, have biomechanical or functional [47] spinal ‘lesions’, but what *else* might they have [48, 49]? Clearly, with aging populations, a significant proportion of people will have multiple health issues which will require management decisions including co-management.

### Information for patients

Chiropractors in common with all other health professionals have an obligation to provide patients sufficient information to allow them to determine what is for them the best course of management [50–52]. Patients, in the end, not health professionals, determine the actions they will take with respect to their own health and illness, including when, how, and from whom they seek care, and how they pursue the recommendations of their various care providers [51]. Leask [53] emphasises information should match people’s conceptual pathways, explain choices and their implications, demonstrate balance, communicate risk in understandable formats and help patients clarify what is important.

## Methods

### The decision-aid

**Fig. 1:**
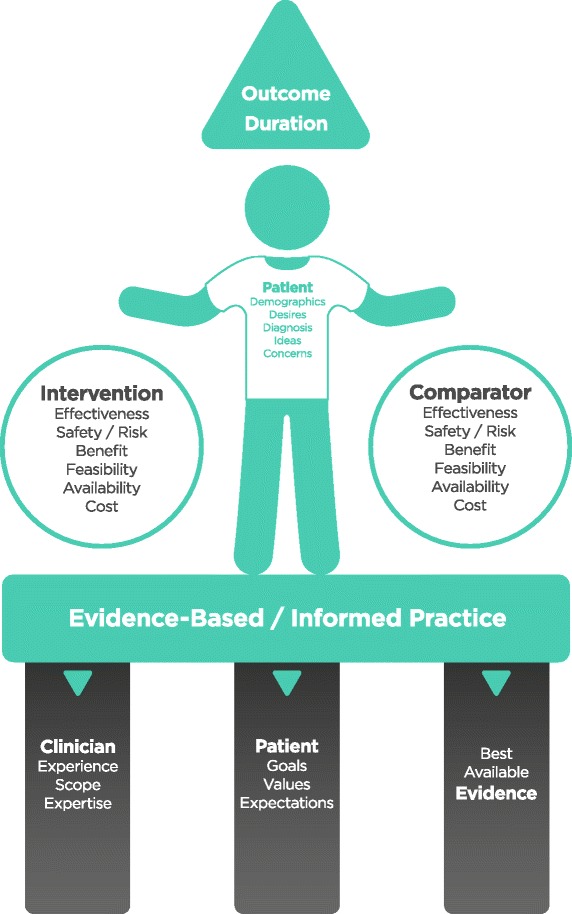
The ‘PICO-D Man’ schematic clinical and educational chiropractic decision-aid for field practitioners and students

This is our decision-aid that can be used in both a teaching and clinical context that facilitates a comparison of evidence and other factors to assist in clinical decision-making. Figure 1 is a schematic representation of the contextual implementation of the classical EBP process of weighing up information that needs to be converted into answerable *questions,* finding the *best evidence* with which to answer those questions, critically *appraising* the evidence for its validity and usefulness, *applying* the results of the appraisal into clinical practice, and finally, *evaluating* performance [54].

This decision-aid involves the clinician applying four simple steps; *describing* the patient and their problem, *comparing* the proposed intervention with feasible options for *this patient* in terms of best available evidence, risk vs. benefit, cost, feasibility / availability, deciding the appropriate way to *measure* the outcome and quantifying the *time*-frame [55, 56]. The variables are effectively weighed much like a set of scales [57].

For example, a likely question for a chiropractor may be framed like this;

*“For a patient with non-specific, acute [or subacute/chronic] low back pain* ***[P],*** *is usual chiropractic care (UCC) composed of active care (discouraging bed rest, providing education, reassurance, addressing fear avoidance, advising activity), and passive care (CSMT and maybe other adjunctive manual methods)* ***[I],*** *at least as effective with comparable risk and cost compared to usual medical care (UMC) and superior to natural history (NH)* ***[C],*** *in reducing pain and improving function* ***[O]*** *over a specific time-frame (ie;12 weeks)* ***[D]*** *?”*

Now the question has been framed, how does one consult the evidence in answering the question? This is a process of starting at the higher levels of the evidentiary hierarchy and working through until the best available evidence is found to apply in the context of the framed question. For the example, with respect to the example of non-specific acute and subacute low back pain, there are readily accessible contemporary clinical practice guidelines that have direct relevance for chiropractors [58].

**Fig. 2:**
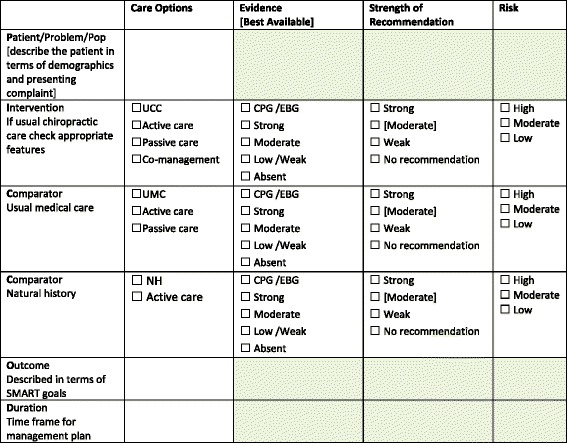
A table for applying the PICO-D Man decision-aid in clinical practice

The quantitative hierarchy of evidence for interventions is well articulated in the literature, the levels of evidence in this space being relevant to informing clinical interventions [59]. Then there is the increasing recognition of the value of qualitative and mixed methods studies; methodologies that ensure the voices of consumers and practitioners are heard, considered and contextualised [60, 61]. A useful table for recording these variables and options is offered in Fig. 2.

We will now review the application of the ‘*PICO-D Man’* decision-aid to a hypothetical example of acute and subacute low back pain.

*Clinical scenario;* A 28 year old male Caucasian carpenter presents with severe low back pain located over the right side of his low back region (he points to the right SI-J). It has been ten days since he reached under a bench for a jig-saw and felt a sharp pain which he now rates as a 7/10 on a numerical rating scale. It is not getting better, in fact if anything it is getting worse. He denies any radiations or pain on coughing or exertion, but it ‘grabs’ him when he twists to the right or looks upwards. He has had a couple of previous episodes about two years ago when he saw another chiropractor for a few weeks and has had no trouble since, however this time the pain also seems to travel up along his spine to the base of his neck. He has not seen anyone else about this pain and is not taking any medications except over the counter pills. He has never had any surgeries or hospitalisations and to his knowledge no family history of serious illnesses or back pain. He is an otherwise healthy male individual who is in obvious distress due to the pain. On examination; vital signs/reflexes/myotomes/dermatomes are all within normal limits. He has a low right iliac crest and left shoulder, muscle guarding over right Quadratus Lumborum, -ve Straight Leg Raise/Bilateral Leg Raise/Slump Test, -ve Valsalva, -ve Fabere-Patrick/Fig. 4, +ve R SI Thrust, Gaenslens, SI compression and distraction tests. Static and motion palpation of the spine and pelvis reveal significant findings in right SI-J. In addition, there are signs of spinal dysfunction affecting the upper thoracic and lower cervical spinal regions.

*Diagnosis;* acute, moderate, right sacro-iliac biomechanical dysfunction (acute low back pain) with associated cervico-thoracic spinal dysfunction (Primary dx: ICD code: M54.5).

#### Problem: acute and subacute low back (spinal) pain

*“What is wrong with me, can you get rid of this pain?”;* the patient description; diagnosis, desires, ideas, concerns and expectations.

#### Best evidence: clinical practice guidelines (CPG)

The field chiropractor can now readily refer online to contemporary CPGs for spinal pain [62, 63]. For example we direct readers to the Canadian Guideline Initiative; (http://www.chiropractic.ca/guidelines-best-practice/) [58].

Referring to contemporary guidelines for acute and subacute low back pain yields the following ‘generic’ recommendations;

- *Education*: Provide advice and information addressing unhelpful beliefs, fear avoidance and to promote self-management.
- *Physical activity*: advise patients with acute and subacute low back pain to stay active and continue activities of daily living within the limits permitted by their symptoms. Discourage prolonged bed rest.
- *Exercise*: should be recommended to reduce the recurrence of low back pain.

*Heat* may be used for pain relief.

#### Intervention: usual chiropractic care (UCC)

*“Can you help?”*

Consider offering a course of manual care, including spinal manipulation over a period of up to 12 weeks in addition to the generic management already outlined [58, 64].

#### Comparator: usual medical care (UMC)

*“What are my options?”*

*Simple analgesia* (e.g. acetaminophen/paracetamol): is routinely recommended in guidelines for acute and subacute low back pain [65]. Recent evidence has however raised serious questions concerning effectiveness [66] and safety [67]). NSAIDs: may also be used for short-term pain relief. *Opioids:* cautious and responsible use of opioids may be considered for those *carefully selected* patients with severe acute pain not controlled with acetaminophen and NSAIDs at a minimum effective dose for a limited period of time, usually less than one to two weeks [68].

### Other interventions

Massage, Acupuncture, Yoga, and Cognitive-Behavioural Therapy are recommended as an option for subacute and chronic LBP [63]. However electro-therapeutics such as ultrasound, TENS and short-wave diathermy are not recommended due to their unknown effectiveness [65].

### Other factors considered within guidelines*

(*We have extracted relevant specific citations from clinical practice guidelines for risk, cost, feasibility, measures and duration).

#### Comparator: natural history

*“What if I do nothing?”*

When considering the natural history of spinal pain it must be noted that there is wide variation in the literature, with up to 80% chance of recurrence and up to a 30% chance of non-resolution inside a year [69]. For patients with multiple regions of pain, the prognosis is even more bleak [70]. Thus, acute back pain can be considered neither as automatically self-limiting nor insignificant. Natural history may possibly be described as “*the future will likely reflect the past. If you have had episodes of pain in the past, recurrence is common*”.

Risk: *“Is it safe?”*

Serious direct adverse events following generic active lifestyle and behaviour modification and self-care recommendations are predictably rare since they essentially relate to natural history [71–73] and the main risk associated with natural history is progression to chronicity [74, 75]. Chiropractic spinal manipulation for acute and subacute low back pain is also rarely associated with serious adverse outcomes, we could locate no documented cases of mortality from low back spinal manipulation and even mild adverse events have not been shown to be more common than placebo [76, 77]. Pharmaceutical management however, *is* however associated with significantly greater risk even for simple analgesia, especially in children and vulnerable populations [78, 79]. Persons with one or more GI risk factor (longer duration of use, higher dose, age 60 years or older, history of peptic ulcer disease, alcohol use, concomitant use of corticosteroids or anticoagulants, or general frailty) should either lower their NSAID dose, take the drugs intermittently - or not to take them at all [80–82]. NSAID use is associated with an increased risk of death or myocardial infarction by up to 5 times that of non-users [83]. Acetaminophen (paracetamol) even taken in low doses by pregnant women is associated with side-effects including higher incidence of ADHD and is the cause of poisoning in 8000 Australians per year [84–86]. Opioid drugs and benzodiazepines are associated with significant risks and complications related to tolerance (and escalating doses), addiction, misuse, abuse and death, particularly with chronic or recurrent spinal pain syndromes. Notwithstanding, some clinical guidelines recommend the judicious use of strong analgesics and benzodiazepines, such as tramadol, oxycodone and diazepam, in acute cases of back pain of less than 4 weeks duration, even though the supporting research evidence is weak [87].

Cost and feasibility: *“How much does it cost?”*

Feasibility and practicality; “*is the proposed plan of management available and within the means of the patient?”* Practitioners must bear in mind that they are acting in effect as the patients’ ‘agent’. There is usually a direct financial benefit that accrues to the practitioner following recommendations, thus there is the issue of the ‘agent moral hazard’ of which to be mindful [88]. Regulations govern the appropriate scheduling and degree of servicing in plans of management [50]. Current data support the management of spinal pain with an approach that includes spinal manipulation and chiropractic compares favourably in cost effectiveness studies [89–91].

Outcome measures: *“How will we measure improvement?”*

All plans of management should include outcomes, milestones or goals. Goals that are focused on function set a more meaningful and holistic target to work towards than those focused on impairments. Management is clinically justified when it promotes independence, improves function and participation, or demonstrably prevents the person from significantly deteriorating from their current level of function [18, 69, 92]. Goals and outcomes should ideally be framed in a SMART format. This acronym is commonly used in many contexts including healthcare. *Specific*; names the particular variable of interest, e.g. the distance the patient is able to walk, or hours at work, *Measurable*; there is a measurement unit (e.g. metres, hours, 0-10 scale), *Achievable*; the goal is likely to be achieved given the diagnosis and prognosis and any environmental constraints, *Relevant;* the goal is important to the person and other stakeholders and there is a *Time-*frame; within which the goal is expected to be achieved. These “outcome measures” thus are framed in terms of the subjective, objective and patient specific (activities of daily living) measures. There are reliable, validated (questionnaire) outcome measures freely available that may be used such as; Revised Oswestry Back Pain Disability Questionnaire and the Quebec Back Pain Disability Scale [93, 94].

#### Duration/timing

Current guidelines recommend plans of management for acute and subacute low back pain be framed with a duration no longer than 12 weeks with appropriate milestones [64].

### Summary

Using our hypothetical patient as an example, the chiropractor is able to cite moderate level evidence and strong recommendations for all the active and passive components of the clinical encounter. If usual medical care is considered as the comparator option, (and assuming it is guideline concordant) one finds moderate evidence and weak recommendations for the medication component [68, 95]. It should be pointed out that *no* intervention for spinal pain has greater than a moderate level of evidence; moderate evidence is thus the *best available* [62].

## Results and Discussion

The application of EBP may seem an insurmountable and complex challenge to the field practitioner due to many factors. Among other well described barriers is the sheer volume of published material against a backdrop of the human propensity to ‘cherry pick’ the evidence to find that which supports one’s preferred methods which leads to a situation in virtually all professions where proponents despair about the low adoption of, and field resistance to EBP [12, 96]. When ‘*PICO-D Man’* is applied in a pragmatic and feasible way, we feel these ‘barriers’ may become ‘enablers’ for the chiropractic profession as clinicians and patients are both empowered by evaluation and application of evidence. Reasoned debate around the issue of adoption of evidence in clinical practice (EBP) *must* include the evaluation of the options available to consumers (comparator). At best, it is incomplete to consider only the proposed intervention side of the equation. In the chiropractic context, evaluation of effectiveness of usual chiropractic care intervention is almost meaningless if not accompanied with both that of usual medical and other care options including the natural history for the condition. When neither intervention is shown to be clinically superior, patient preference becomes the salient variable. Preference-sensitive treatment decisions involve making value trade-offs between benefits and harms that should depend on informed patient choice. There is strong evidence that patient decision-aids not only improve decision quality but also prevent the overuse of options that informed patients do not value [97].

### Implementation

We are of the view that being overly critical of field practitioners for low adoption of EBP is not productive and thus propose an approach that entails education via demonstrating that EBP is actually in everyone’s best interest, including practitioners. We recognise there is continuing debate over whether EBM is markedly better than standard teaching methodologies; although some evidence for the effectiveness of EBM has accumulated, there is still only emerging evidence on what are the most effective methods of teaching it [98–100]. There is also still an ongoing robust debate as to whether practitioners of EBM actually provide better health care [101] than those who do not [102–107]. Just as clinicians recognise that chastising risky behaviour by patients will not alter it, so with field clinicians. Tying desired changes to existing norms helps people understand and adopt practices, as does how messages are framed [12].

It must be highlighted that usual care by any health professional, including chiropractic, is a package not one treatment in isolation; *chiropractic is a profession, not a technique*. Management of patients entails a complex combination of variables that make each and every person unique, a so called random sample of *n* = 1 [108]. Discussions regarding clinical management must take account of the entire clinical encounter rather than focussing on one isolated aspect [109]. However stakeholders including third party payers and administrators usually require that clinical decision making by health care providers should rely as far as is possible on evidence deriving from research, which may be practice-based [110]. ‘*PICO-D Man’* directs attention to the clinical application in a way that is relevant to chiropractors including possibly in the context of conducting practice-based research [111]. Some chiropractors, in our experience are fond of the saying; *“Chiropractic works; it gets results and that’s what counts”;* but it may reasonably be countered with “*compared to what and by how much and for whom”? ‘PICO-D Man’* represents how that question may be addressed and EBP sensibly applied in a clinical context. While this paper presents the example of a person with acute or subacute spinal pain the model may find application for other clinical scenarios [112].

It is important to point out the clinician has a responsibility to develop a management plan that is *defendable,* not one that is necessarily agreed to by all other health care professionals. Adherence to CPGs while relatively higher among chiropractors, is a challenge for all primary care professions in the spinal care sector [113]. Patients generally have realistic expectations, although some may consider their prognosis to be different from that which would be expected from known prognostic factors [114]. Accordingly, clinicians should pay attention to previous experience in patients with low expectations rather than focusing on psychological factors such as depressive symptoms and fear avoidance beliefs [114]. It should also be remembered there may also be a genetic predisposition to LBP [115] which highlights the importance of obtaining a family history about back pain.

Triano [116] provided a helpful guide based on the positions of Sackett [117, 118] and Sox [119] in recommendations for provider considerations when strong evidence is absent; i.e. Review available evidence, is it physiologically plausible? Is the thinking is based on valid evidence? Consider costs to the patient, when in doubt, take special care to avoid actions that might cause harm, whether it is physical, emotional, or economic. Above all, *talk* to the patient, explaining the ambiguity in the evidence and the steps proposed [116]. In clinical circumstances where there is low or absent evidence, a three question ‘Traffic Light System’ can also be useful as a simple framework to help chiropractors make clinical decisions in a simple and lucid manner; Are there objectively tested facts to support the concept? Are the concepts that form the basis for this clinical act or decision based on other scientifically acceptable concepts? and Is the concept based on long-term and widely accepted experience [120]?

The simplest approach to a problem can usually be defended. Chiropractic is in essence a straightforward system of clinical management; *rule out* serious causes, e*ducate* the patient regarding spinal health and self-care, promote *healthy lifestyle* choices (diet, rest, physical activity, positive mental attitude) and provide *manual care* as appropriate.

### Strengths and limitations

The extent to which this decision-aid may assist in clinical practice still needs to be tested; thus it is presently used in the educational domain where anecdotally it appears useful. The field of ‘translational research’ which explores the implementation of EBP in healthcare is complex. As Kessler and others highlight; *“complex problems of complex patients embedded in complex healthcare systems in complex and changing communities require complex interventions”* [121–124]. In the scenario offered here, we have presented the illustration of acute/subacute spinal pain and provided the reader with *one* answer in this context; this application of the algorithm leads to *one* answer, and because of its flexibility allows for the cultural, social and other perspectives (of both patients and providers) to lead to individualised decision-making. While we feel this is the strength of the decision-aid, it does not replace the specific needs of all practitioners who will invariably have unique cultural and social overlays respecting their patients in their own clinics. Another limitation that should be mentioned is the changing nature of the evidence and the need and challenge for practitioners to stay up-to-date. Information contained in guidelines may be out-dated by the time they are published. Responsible clinicians have to be cognizant of this and thus cannot blindly follow guidelines without question. Translating a *culture* of EBP into everyday clinical practice is a challenge and while we feel our approach is simpler and more intuitive than other more complex Critical Appraisal Skills Programme (CASP) sheets [125] such as the Graphic Appraisal Tool for Epidemiologic Studies (GATE Frame) [126], this position would need to be established via a formal reliability/validity study. Evaluation studies have shown that decision aids improve decision making in many ways including increasing patient participation in decision making without adversely affecting anxiety [127, 128]. We feel an ideal platform to investigate the feasibility of the tool would be using a established Australian Practice-Based Research Network (ACORN) in collaboration with experienced ACORN methodologists [129]. Field practitioners may possibly be more likely to use a decision-aid if it can be shown to streamline their clinical practice and facilitate (for example) third party payments possibly via an online or mobile device ‘*PICO-D Man’* ‘app’.

## Conclusion

The decision-aid is offered here in the hope it may prove useful to educators and field clinicians alike in applying evidence-informed practice within the chiropractic profession. Academics, clinicians, researchers, students and patients often view the health-care world and the place of chiropractic in it in drastically different ways. Thus, this paper is partly aspirational and is presented principally to assist chiropractic students, educators and field practitioners in ethical and defensive, contextual clinical decision-making. Students often appear unable to integrate the principles of EBP they have learned in the academic curriculum in a practical clinical setting, and field practitioners frequently express an overt disinterest or outright antithesis toward EBP, a phenomenon by no means confined to the chiropractic profession. For students, one collateral effect of engaging in a formal ‘application’ process is often enhanced confidence in clinical management-plan construction, and for field practitioners, (for example) justification for payment. With integration into informed consent, patient feedback and possibly simulated learning and ongoing learning (CPD) scenarios; the decision-aid could conceivably inform research and further facilitate evidence-informed clinical practice. The decision-aid may also help clinicians provide healthcare consumers with patient-centred care.

## Abbreviations

ADHD: Attention deficit hyperactivity disorder
CPD: Continuing professional development
CPG: Clinical practice guideline
EBCP: Evidence-based chiropractic practice
EBG: Evidence-based guideline
EBP: Evidence-based practice
GI: Gastro-intestinal
LBP: Low back pain
NH: Natural history
NSAIDs: Non-steroidal anti-inflammatory drugs
SMART: Specific measurable achievable relevant timed (Goals)
UCC: Usual chiropractic care
UMC: Usual medical care.
